# Barnacle Geese Achieve Significant Energetic Savings by Changing Posture

**DOI:** 10.1371/journal.pone.0046950

**Published:** 2012-10-10

**Authors:** Peter G. Tickle, Robert L. Nudds, Jonathan R. Codd

**Affiliations:** Faculty of Life Sciences, University of Manchester, Manchester, UK; University of Roehampton, United Kingdom

## Abstract

Here we report the resting metabolic rate in barnacle geese (*Branta leucopsis*) and provide evidence for the significant energetic effect of posture. Under laboratory conditions flow-through respirometry together with synchronous recording of behaviour enabled a calculation of how metabolic rate varies with posture. Our principal finding is that standing bipedally incurs a 25% increase in metabolic rate compared to birds sitting on the ground. In addition to the expected decrease in energy consumption of hindlimb postural muscles when sitting, we hypothesise that a change in breathing mechanics represents one potential mechanism for at least part of the observed difference in energetic cost. Due to the significant effect of posture, future studies of resting metabolic rates need to take into account and/or report differences in posture.

## Introduction

When evaluating energy budgets in birds, few studies have taken into accounts any differences in the cost associated with different postures. Many birds spend a significant proportion of time resting [1]. However, while resting metabolic rate has been measured in a wide range of birds [2], [3], the effects of posture upon metabolism were only previously considered in three studies of domestic fowl [4], [5] and two studies of guillemots [6], [7]. One of these [4] estimated a 42% increase in metabolic rate when standing compared to sitting, but this value included the cost of rising. The study of Van Kampen [5] suggested the metabolic cost associated with just standing was 16% (25% if the fowl were also indulging in spontaneous pecking and preening behaviour) greater than sitting. Ellerby *et al.* [8] concluded that standing was more metabolically costly than sitting without quoting exact values. Compared to a lying prone posture, standing upright incurs a metabolic rate increase of between 7% and 9% in guillemots [6], [7], lower than the values observed in fowl. Environmental conditions, such as temperature, exposure to sunlight and precipitation, are regarded as the main factors affecting posture selection [9], [10]. It is difficult, however, to disentangle whether any concurrent change in metabolic rate is due to a homeostatic response, such as temperature regulation [11], or simply the energy cost of maintaining a particular resting posture. Selection of a resting posture might also be affected by the critical requirements of breathing. Respiratory muscles actively move the rib cage and sternum during ventilation, a process that can be constrained by posture [12]. Therefore, knowing the metabolic cost of sitting and standing in birds is important, because it may shed light upon posture selection and behaviour in birds. In addition, posture dependent metabolic costs would have strong implications for studies of resting metabolic rates. Specifically, which posture was adopted during measurements of resting metabolic rate and does that posture really represent the least energetically expensive and therefore, truly resting metabolism? Given its far-reaching implications, it is surprising that so few studies have considered the metabolic costs of different resting postures. Accordingly, in this paper we begin to address this dearth in posture studies by presenting data on the resting energetics of barnacle geese (*Branta leucopsis*) during sitting and standing under constant environmental conditions. Our data augments that currently available and expands it into another phylogenetic order (i.e. the Anseriformes).

## Materials and Methods

### (a) Animals

Barnacle geese were kept at the University of Manchester, housed in social groups and provided with free access to a pond. Food (Poultry Grower Pellets, Small Holder Range, Norfolk, UK: fat 4.8%, protein 16%, carbohydrate 73.7%, fibre 5.5%) and water were provided *ad libitum*. Goose body mass was 1.79 kg±0.03 (mean ± SE). All experimental procedures were covered under a Home Office Licence held by J.R.C. (40/3001) and the ethical approval of the University of Manchester.

### (b) Respirometry

Indirect calorimetry via flow-through respirometry was used [8], [13]. Air was drawn from the respirometry chamber (volume: 148 L) at a rate of 100 L min^−1^. This relatively high flow rate meant changes in gas composition were quickly detected (ca. 20 s). Water vapour content of subsampled (100 mL min^−1^) excurrent air was measured using an RH-300 humidity meter (Sable Systems, Las Vegas, NV, USA) after which the air stream was dried by passing through a column of magnesium perchlorate (Acros Organics, NJ, USA). A CA-10a carbon dioxide (CO_2_) analyser (Sable Systems, Las Vegas, NV, USA) then recorded CO_2_ content of the sample air.

Resting metabolic rate was measured as the rate of carbon dioxide production (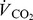) and was calculated using Equation 10.5 from Lighton [14]:
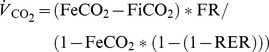
(1)where FiCO_2_ and FeCO_2_ are the concentrations of carbon dioxide flowing into the respirometry chamber and after leaving the chamber, respectively. FR is the flow rate of air into the chamber after mathematical correction for the presence of water vapour (using Eq. 8.6 of Lighton [14]) and RER is respiratory exchange ratio (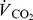: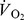). It is not clear how resting posture may affect RER since the underlying physiological mechanisms are not fully understood [15]. Therefore, since oxygen consumption (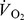) was not measured in these experiments, an RER of 0.85 was assumed in order to minimise error in the subsequent metabolic calculation [16]. An RER of 0.85 is also consistent with previous reports of resting metabolism in the barnacle goose (Nolet [17]: 0.77; Nudds [18]: 0.79; P. G. Tickle (unpublished data): 0.87). 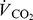 was converted to mass specific power (W kg^−1^) using the thermal equivalents in table 12.1 of Brody [19].

### (c) Experimental procedure

Prior to the trials presented in this paper, geese had been used for separate experiments [13], [18]. Consequently, these birds were very familiar with being housed inside the chamber and showed no discernable stress during the sitting and standing experiments. The temperature inside the chamber (20.1°C±0.2) was maintained within their thermoneutral zone [20]. Trials were conducted in daylight hours and lasted 121.3±9.2 minutes (mean ± SE). By standardising laboratory conditions (temperature, light intensity and humidity) we controlled for factors that can potentially influence choice of posture [9]. Food was not provided within the respirometry chamber. Straw bedding, however, identical to that found in the housing area, was placed inside the respirometry chamber to encourage natural resting behaviour. During each trial the goose was allowed to walk into the respirometry chamber and then left alone in the experimental room. Behaviour was monitored remotely using a webcam connected to a computer that was programmed to take a photograph at 10-second intervals. Goose posture was then established from the photographs and synchronised to the corresponding respirometry data. Steady periods of 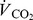 stable for at least 180 seconds, after accounting for the time difference (ca. 20 s) between instantaneous behaviour record and detection in the analyser), corresponding to sitting and motionless standing posture were considered representative of resting metabolism. In addition, the different postures were displayed in no particular order.

### (d) Data analyses

Data from a total of 17 trials (each trial represents data from 1 bird) using 10 birds were analysed in this paper, representing a larger sample size than used in previous studies of postural energetics [4], [5], [6], [8]. In all cases (sitting and standing), data was taken from the trace as the 3-minute period with least variation in 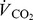. A repeated measures ANOVA was used to test whether posture (sitting or standing) and the individual goose (random factor) affected 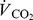. All means are displayed as ± standard error. The statistical analyses were performed using the statistics toolbox in MATLAB® R2007b (The MathWorks, Inc., 3 Apple Hill Drive, Natick, MA).

## Results

**Figure 1:**
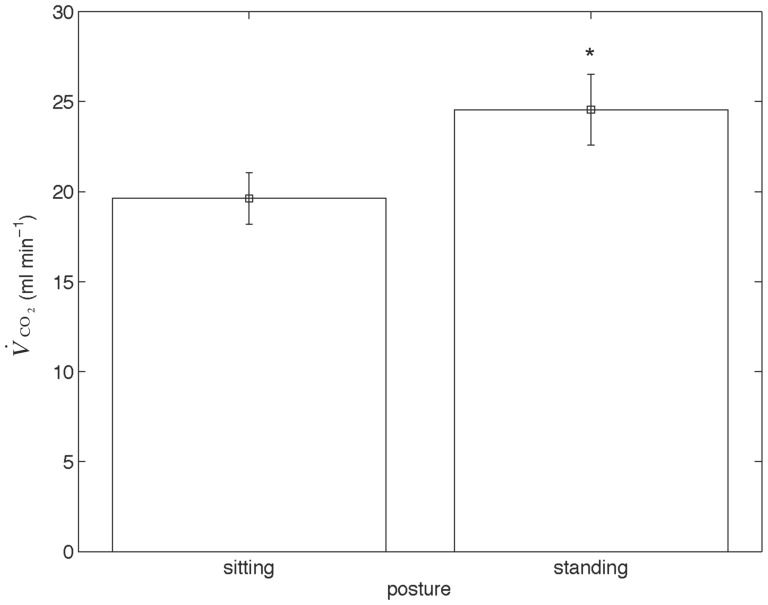
The energetic effect of resting posture in barnacle geese. Rate of carbon dioxide production (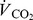) plotted for the barnacle geese during sitting and standing. The 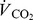 of the geese was higher when standing (24.55 ml min^−1^±1.96) than sitting (19.62 ml min^−1^±1.44), indicating that sitting is metabolically cheaper.

Standing was 25% more metabolically costly than sitting (Fig. 1) and 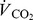 was also affected by the individual goose (posture, *F*_1, 23_ = 9.54, *r* ^2^ = 0.11, *p* = 0.005; goose, *F*_9, 23_ = 5.65, *r* ^2^ = 0.61, *p*<0.001). There was no interaction between goose and posture (*F*_9, 14_ = 0.43, *p* = 0.899) so this interaction term was removed from the final two-way ANOVA described in the preceding sentence. Converting 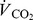 to metabolic power yields 5.50±0.46 Wkg^−1^ for standing and 4.41±0.36 Wkg^−1^ for sitting, both lower than the 6.73 Wkg^−1^ estimated of a zero walking speed (i.e., standing cost) by Nudds *et al.* [18].

## Discussion

Here we demonstrate a significant effect of posture upon resting metabolic rate. The observed increase in metabolism associated with standing is consistent with an earlier report in guinea fowl [8], guillemots [6], [7] and domestic white leghorn hens [5]. The magnitude of the increase here (25%), however, exceeds the 16% reported for domestic white leghorn hens [5] and 7–9% in guillemots [6], [7]. A 25% increase in metabolic costs of standing over sitting was found when the leghorns were not settled and were indulging in extraneous activities (e.g., pecking or fluffing out their feathers). The geese in this study did not perform any auxiliary behaviour when standing in the respirometry chamber. Therefore, it is likely that the increased costs in the geese are due to morphology. Specifically, the barnacle geese have large pectoralis and supracoracoideus muscles to permit sustained flight, equivalent to 17.6–17.8% body mass [21]. White leghorn chickens have comparatively smaller flight musculature, accounting for 12.3% of total body mass [22], while guillemot breast muscle equates to 10.4–11.1% of body mass [23]. The extra weight upon the sternum in the goose is likely to increase the cost of respiration when standing, because it must be moved up and down with the sternum during a breathing cycle [12].

The statistical effect of individual goose on the magnitude of postural dependent change in metabolism is intriguing. As the birds were not fasted prior to experimentation it may be that the postprandial energetic costs [19] varied according to previous feeding behaviour [24]. Interspecific analyses often use criteria to ensure common experimental procedures have been implemented across data sources, such as the maintenance of species-specific thermoneutral zones and circadian rhythms, since these factors can affect resting metabolic rate [2], [3]. In light of our experiments, careful consideration should be given to how resting posture could influence estimates of basal and resting energy metabolism.

Research on terrestrial locomotion in birds has identified a discrepancy between projected resting metabolism, calculated by extrapolating the straight line relationship between speed and metabolism, and measured resting rate [25]. This is assumed to represent the cost of maintaining posture [25]. Our calculations of the resting cost for standing and sitting are both lower than the metabolic rate estimated in Nudds *et al* [18] (standing is 18.3% lower, sitting is 34.5% lower), indicating a significant metabolic effect of stress and alertness associated with treadmill locomotion. Our results support the hypothesis that experimental stress represents an alternative mechanism to account for the high rate of resting metabolism when derived from walking/running costs [26].

By definition, measuring energy consumption in the thermoneutral zone eliminates the potential for increased metabolic activity associated with thermoregulation. What then are the metabolic processes that account for the disparity between standing and sitting postures? Constant muscle activity is required to maintain balance and posture; fatigue-resistant slow muscle fibres are found in muscles around the hip and knee to maintain the crouched leg standing posture in birds [27]. While it is very likely that a proportion of the metabolic disparity can be accounted for by the reduced hindlimb muscle activity during sitting, calculation of the muscular cost of standing is constrained by lack of data from resting birds. For example, the force produced in postural leg muscles during isometric contraction is unknown and a study of metabolism at the level of individual muscles is available only for guinea fowl [28]. Any attempt to partition the metabolic cost of posture into its constituent parts remains speculative.

Interestingly, ventilatory mechanics in birds depend upon posture. When standing, breathing involves dorso-ventral rotations of the sternum [12], [29]. In contrast, ventilation is maintained by lateral excursions of the rib cage when sitting [12], [29]. It follows that the drop in energetic cost of breathing may be a result of sitting and therefore not having to move the large mass of the sternum to breathe. Previous attempts to quantify the energetic cost of breathing have indicated that only around 2% of whole organism metabolism is dedicated to maintaining ventilation [28], [30]. A recent study of load-carrying energetics in barnacle geese however indicated that when compared to unloaded trials, geese with artificially increased sternal mass were often found to rest lying down and walking locomotion was more energetically costly [13]. Based upon this study [13] and the results presented here we hypothesise that the relatively inexpensive sitting posture may be in part accounted for by economical breathing energetics. Moreover, differences in pectoralis muscle mass loading of the sternum driven by the locomotor needs of a bird species (e.g., predominantly terrestrial locomotion in Galliformes and flight requirements in Anseriformes) are likely to result in profound differences in the metabolic costs of standing versus sitting. The driving of standing energy costs by sternal loading also has implications for the physiology and behaviour of species that undergo premigratory hypertrophy of flight muscles [21], [31] and domesticated species that are selected for large pectoral muscle mass. The latter could result in a situation whereby the respiratory system is compromised and as a consequence, the welfare of the animal too.
